# Evaluation of a machine learning tool for the early identification of patients with undiagnosed psoriatic arthritis – A retrospective population-based study

**DOI:** 10.1016/j.jtauto.2023.100207

**Published:** 2023-08-02

**Authors:** J. Shapiro, B. Getz, S.B. Cohen, Y. Jenudi, D. Underberger, M. Dreyfuss, T.I. Ber, S. Steinberg-Koch, A. Ben-Tov, Y. Shoenfeld, O. Shovman

**Affiliations:** a Maccabi Healthcare Services, Israel; b Predicta Med Analytics Ltd, Israel; c Metroplex Clinical Research Center, Dallas, TX, USA; d Maccabi Institute for Research & Innovation, Maccabi Healthcare Services, Tel Aviv, Israel; e Sackler Faculty of Medicine, Tel Aviv University, Tel Aviv, Israel; f Zabludowicz Center for Autoimmune Diseases, Sheba Medical Center, affiliated with Tel-Aviv University, Israel; g Department of Medicine B, Center for Autoimmune Diseases, Sheba Medical Center, Tel Hashomer, Israel

**Keywords:** Psoriatic arthritis, Machine learning, Artificial intelligence, Psoriasis, Early diagnosis

## Abstract

**Background:**

Psoriatic arthritis (PsA), an immune-mediated chronic inflammatory skin and joint disease, affects approximately 0.27% of the adult population, and 20% of patients with psoriasis. Up to 10% of psoriasis patients are estimated for having undiagnosed PsA. Early diagnosis and treatment can prevent irreversible joint damage, disability and deformity. Questionnaires for screening to identify undiagnosed PsA patients require patient and physician involvement.

**Objective:**

To evaluate a proprietary machine learning tool (PredictAI™) developed for identification of undiagnosed PsA patients 1–4 years prior to the first time that they were suspected of having PsA (reference event).

**Methods:**

This retrospective study analyzed data of the adult population from Maccabi Healthcare Service between 2008 and 2020. We created 2 cohorts: The general adult population (“GP Cohort”) including patients with and without psoriasis and the Psoriasis cohort (“PsO Cohort”) including psoriasis patients only. Each cohort was divided into two non-overlapping train and test sets. The PredictAI™ model was trained and evaluated with 3 years of data predating the reference event by at least one year. Receiver operating characteristic (ROC) analysis was used to investigate the performance of the model, built using gradient boosted trees, at different specificity levels.

**Results:**

Overall, 2096 patients met the criteria for PsA. Undiagnosed PsA patients in the PsO cohort were identified with a specificity of 90% one and four years before the reference event, with a sensitivity of 51% and 38%, and a PPV of 36.1% and 29.6%, respectively. In the GP cohort and with a specificity of 99% and for the same time windows, the model achieved a sensitivity of 43% and 32% and a PPV of 10.6% and 8.1%, respectively.

**Conclusions:**

The presented machine learning tool may aid in the early identification of undiagnosed PsA patients, and thereby promote earlier intervention and improve patient outcomes.

## 1 Introduction

Psoriatic arthritis (PsA), a chronic inflammatory form of arthritis leading to joint damage, occurs in ∼20% of patients with psoriasis (PsO), with an equal prevalence in males and females [1].

The reported incidence of PsA among psoriasis patients is between 0.27 and 2.7/100 person-years [[2], [3], [4], [5]]. The majority of patients initially present with skin PsO [6], with arthritis developing up to 20 years after the first PsO diagnosis; however, arthritis may precede skin involvement or occur concurrently [7].

PsA incidence and prevalence in the general population vary widely, and primarily depend on geographic location, and may be rooted in differences in genetic background, as well as the quality and accuracy of registries and databases. The incidence ranges between 6 and 8/100,000 person-years in most European countries [8] and is estimated at 10.9/100,000 person-years in the adult population in Israel [9]. PsA prevalence is estimated in a range between 0.1 and 1% of the general population, and 0.15% in the adult Israeli population [[7], [8], [9]]. When based solely on epidemiological studies, the prevalence of undiagnosed PsA among PsO patients is approximated at 10.1% [10,11]. Yet, the true prevalence of undiagnosed PsA in the general population is currently unknown.

The median time from symptom onset to PsA diagnosis is 2.5 years, with only 45% of patients diagnosed within 2 years [12]. Early diagnosis of PsA is essential, since as little as a 6-month delay in treatment initiation may lead to peripheral joint erosions [13], irreversible physical disability and deformity, and an overall reduction in health-related quality-of-life [4,[13], [14], [15], [16], [17], [18]]. Additionally, the association of PsA with cardiovascular comorbidities underlies the importance of early detection [19,20]. The PsA Classification Criteria (CASPAR) requires information from physicians, results of laboratory tests and imaging studies [21]. This has prompted the development of screening questionnaires [[22], [23], [24], [25], [26], [27], [28], [29]] for PsO patients to enable earlier detection of PsA patients [22] and reduce time-to-referral to a rheumatologist [14]. While these screening questionnaires are generally user-friendly, time-effective and highly sensitive and specific, they still require involvement of both the physicians and patients. To date, no computerized machine learning algorithm has been developed and made available to scan large databases to identify undiagnosed PsA patients. Such an algorithm should be able to aggregate and analyze electronic medical record (EMR) data, and inform medical providers of suspected undiagnosed PsA patients.

Here we describe the development of PredictAI™, a clinical decision support (CDS) tool that empowers machine learning models for early identification of patients with a high probability of undiagnosed autoimmune or immune-mediated inflammatory diseases, applied here to focus on PsA. Considering the 2.5-year median time from symptom onset to PsA diagnosis, the performance of PredictAI™ in identifying undiagnosed PsA patients within the primary care setting 1–4 years before the first time they were ever suspected of having PsA (reference event) was evaluated.

## 2 Materials and methods

This retrospective study analyzed anonymized electronic medical records (EMR) of active adult Maccabi Healthcare Services (MHS) members. MHS is Israel's second-largest health maintenance organization (HMO), with ∼2.5 million members, covering 26% of the country's population [12,30]. EMR data were available via the Kahn Sagol Maccabi Research and Innovation Center (KSM). The data included structured demographics, laboratory results, inpatient and outpatient visit reports, body mass index (BMI), diagnoses, procedures and medications.

The data were extracted using MDClone, a big data platform which stores and processes data on a regular basis. The data used were first de-identified by KSM; no identifying details were exposed to the researchers. Ethics approval was obtained from Maccabi Institutional Review Board (IRB; approval #0052-20-MHS).

In the absence of a specific test for PsA that could serve as a marker of initial disease suspicion, the first PsA diagnosis registered by any physician was taken as the reference event while considering the possibility that initial suspicion could have preceded the first registered diagnosis.

## 3 Study population

Two cohorts were created for this study: a general population cohort (“GP Cohort”) that included patients with and without psoriasis and a PsO cohort that included only the PsO patients from the GP cohort. In each cohort, reference dates for subjects without PsA were selected to match the dates of the reference events of PsA patients. For each subject, 3 years of consecutive data collected from one year prior to the date of the reference event were used in the model. For example, if a patient's reference event was dated January 2016, the model was presented with data dating from January 2012 to January 2015.

The GP cohort was comprised of randomly selected adult members of MHS aged 18–84 years, with at least 7 years of consecutive data prior to the reference event, between the years 2008 and 2020. The following case-identifying criteria (CIC) [31] were used to identify PsA and PsO patients:

PsA:

(i) At least 2 PsA diagnoses by a rheumatologist, or
(ii) At least 1 PsA diagnosis by a rheumatologist and 1 psoriasis diagnosis by a dermatologist
  PsA patients diagnosed before 2008 were excluded.

PsO:

(i) At least 1 PsO diagnosis by a dermatologist

Diagnosis codes for PsA and PsO were obtained from the MHS internal coding system that is based on ICD-9 codes and were used to identify PsO and PsA patients (Table 3).

**Table 1:** Sensitivity, PPV and AUC at specificity cutoff points - 99.0%, 99.9% for the test GP cohort.

	Sensitivity %, [95% CI]		PPV % [95% CI]		AUC
	99.0% Spec	99.9% Spec	99.0% Spec	99.9% Spec	
1 year gap	43% [36–49]	17% [12–22]	10% [9–11]	32% [22–37]	89.8%
2 years gap	37% [31–44]	15% [10–20]	9% [8–11]	29% [23–39]	87.3%
3 years gap	33% [26–40]	11% [7–16]	8% [7–10]	23% [15–30]	85.7%
4 years gap	32% [25–38]	11% [7–16]	8% [7–10]	23% [16–32]	84.2%

**Table 2:** Sensitivity, PPV and AUC at specificity cutoff points - 90%, 95% for the test PsO cohort.

	Positives found %, [95% CI]		PPV % [95% CI]		AUC
	90.0% Spec	95.0% Spec	90.0% Spec	95.0% Spec	
1 year gap	51% [42–58]	35% [27–42]	36% [30–39]	43% [35–49]	78.7%
2 years gap	43% [36–51]	31% [24–39]	32% [27–37]	40% [34–49]	75.7%
3 years gap	42% [32–47]	29% [23–37]	31% [26–35]	39% [34–49]	72.8%
4 years gap	38% [30–45]	26% [19–33]	29% [26–36]	40% [32–49]	71.5%

**Table 3:** Diagnosis codes for PsA and PsO from the MHS internal coding system that correspond to relevant ICD-9 codes.

Internal “Ycode"	Diagnosis description	ICD9 code	Modifier
Y16498	PSORIASIS VULGARIS	696	02
Y16493	PSORIASIS	696	01
Y16479	PSORIASIS INVERSE	696.1	05
Y16492	GUTTATE PSORIASIS	696.1	04
Y16496	PSORIASIS PUSTULAR	696.1	07
Y16488	ACRODERMATITIS CONTINUA OF HALLOPEAU	696.1	02
Y16487	PSORIATIC ARTHROPATHY	696.0	00
Y23153	PSORIATIC ARTHRITIS	696.0	01

The machine learning model developed in this research was designed to analyze structured data registered in the EMR, including diagnoses, laboratory results, physician encounters, specialist or hospital visits and referrals, procedures, prescribed and filled medications, clinical measurements, family history, and demographic parameters.

**Fig. 1:**
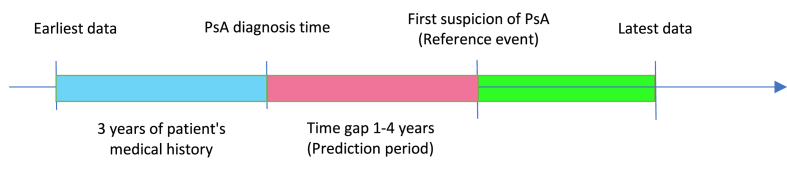
Time gap model for training PredictAI™.

Patient data from the GP and PsO cohorts were each randomly divided into non-overlapping training and test sets. The test sets (15% the size of the training sets) were set aside and were not used for model development. The PsO test cohort was down-sampled so that the *ratio* of undiagnosed PsA patients would be 10% of PsO patients - corresponding to the estimated prevalence of undiagnosed PsA patients among PsO patients [10]. The training sets were used to develop the PredictAI™ algorithms based on 3 years of data predating a one-year time gap prior to the reference event (Fig. 1). The features collected per patient were preprocessed according to the different data types and their statistical distributions, factoring in known correlations to other parameters such as age, sex, height and diagnoses. Counter type features, e.g., yearly frequency of physician visits or prescriptions, were scaled according to age and biological sex statistics. Features with continuous numeric values, such as height, weight, BMI and lab results, were log-normalized to zero mean, unit variance z-scores (based on age and sex-adjusted population statistics) according to the equation:

$$Z=\frac{\log\left(x\right)-\mu }{\sigma }\text{,}$$

where μ,σ are mean and standard deviation, respectively, as a function of a moving average of age and sex.

The cohort data were collected and preprocessed after they were divided into the training and test sets. The training set was used to: (i) study the data, (ii) collect feature statistics, (iii) train the model and optimize its parameters, (iv) perform cross-validation analysis and (v) estimate output variance. The test set was used to test and evaluate the model performance and generate the results presented herein.

The pre-processed EMR data were passed through a machine learning model based on an ensemble of gradient boosted trees (GBT). GBT is a machine-learning technique where several decision trees are fitted to the data in an ensemble of weak classification models. The GBT model handles missing data by default, thus obviating the need to further handling of missing values.

In our test, the PredictAI™ model produced a PsA risk score that was then compared with a calibrated threshold, allowing us to control the trade-off between sensitivity and specificity. We used the threshold to generate plots of sensitivity vs. specificity as well as plots of the positive predictive value (PPV).

The performance of the PredictAI™ algorithm was evaluated using standard measures of sensitivity, specificity, area under the receiver operating characteristic (ROC) curve (AUC) and PPV (%). Sensitivity was calculated as the percentage of correctly flagged (true positives) PsA patients out of all patients meeting CIC for PsA in each cohort. Specificity was calculated as the percentage of correctly non-flagged subjects (true negatives) out of all subjects not meeting CIC for PsA. PPV was calculated as the percent of true positive subjects among flagged subjects.

Plots of ROC analyses (including AUC) present the model's performance at multiple predefined thresholds.

Data was accessed via the MDClone platform, version 5.5.0.4.

All measurements including sensitivity, specificity, PPV and AUC (SSPA) were statistically analyzed using Python Scikit-Learn library version 0.24.2, SciPy library version 1.7.0, NumPy library version 1.20.3^32,33^. Confidence intervals for sensitivity and specificity were calculated using the proportion confidence interval of the binomial distribution. PPV and AUC confidence intervals were calculated via logit-normal approximation

$$logit\left(PPV\right)=\log\left(\frac{PPV}{1-PPV}\right)$$

$$Var\left(logit\left(PPV\right)\right)=\left(\frac{1-Se}{Se}\right)\frac{1}{n_{1}}+\left(\frac{Sp}{1-Sp}\right)\frac{1}{n_{0}}\text{,}$$

where $n_{0}$ is the total number of control patients, and $n_{1}$ is the total number of PsA patients.

The 95% confidence interval for the PPV was calculated as follows:

$$\left[\frac{e^{logit\left(PPV\right)-1.96\;\sqrt{var\left(logit\left(PPV\right)\right)}}}{1+e^{logit\left(PPV\right)-1.96\;\sqrt{var\left(logit\left(PPV\right)\right)}}},\frac{e^{logit\left(PPV\right)+1.96\;\sqrt{var\left(logit\left(PPV\right)\right)}}}{1+e^{logit\left(PPV\right)+1.96\;\sqrt{var\left(logit\left(PPV\right)\right)}}}\right]$$

The PPVs calculated for the PsO cohort reflect the prevalence of undiagnosed PsA in our PsO cohort. A corrected PPV based on different estimates of the prevalence of undiagnosed PsA patients among PsO patients [10] can be calculated as follows:

$$PPV=\frac{Se*Prev}{Se*P+\left(1-Sp\right)*\left(1-Prev\right)}$$

The most prominent features contributing to the identification of PsA were found using the SHAP library (SHapley Additive exPlanations) [32]. The SHAP library calculates the Shapley values (named after J. S. Shapley) for each feature thus enabling to order the features based on their average contribution to the model's results. The GBT algorithm started with 217 features, built a model using approximately 54 of them and using the SHAP library we ordered the features according to their relative contribution to the model and selected the 15 highest influential features (which contributed most to the model's decisions) to be presented in our results.

## 4 Results

### 4.1 Study population

**Fig. 2:**
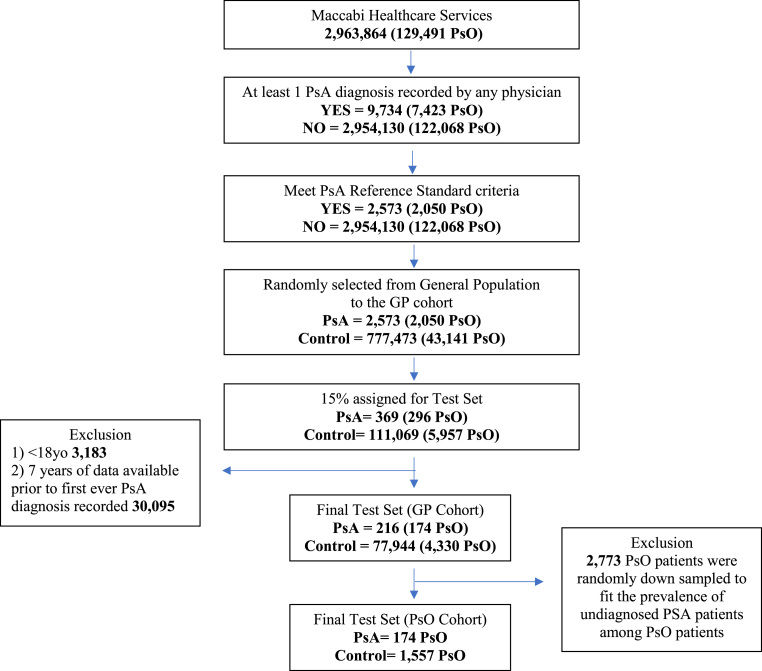
STARD flow diagram.

The STAR-D flow diagram of the cohorts included in this study is presented in Fig. 2. The training set included a total of 640,092 MHS members. The GP cohort included all 640,092 members, among whom 37,953 (5.9%) had PsO and 1880 (0.29%) had PsA. The PsO cohort included these same 37,953 members, among whom 1519 (4%) had PsA.

The test set included 78,160 members. The test GP cohort included all 78,160 members, of whom 4504 (5.8%) had PsO and 216 (0.28%) had PsA. The mean age of the test GP cohort was 45.8 (range: 20–84) years and 52.3% were female.

Number of members, average age and gender of each cohort in the training and test set are presented in Table 4).

**Table 4:** Number of members, average age and gender of each cohort in the training and test set.

		Training set			Test set		
		n	Age (range)	% Male	n	Age (range)	% Male
**PsO cohort**	PsA patients	1511	47.9 (18–83)	47.8	173	49.2 (21–81)	45.6
	PsO patients	36268	47.7 (18–84)	49.3	1557	49.8 (21–83)	49.9
**GP cohort**	PsA patients	1880	48.5 (18–83)	45.9	216	49.9 (21–84)	43.9
	PsO patients	37953	47.7 (18–84)	49.5	4504	49.6 (21–83)	49.1
	Patients without PsO	638212	43.8 (18–84)	47.8	77944	45.8 (20–84)	47.6

### 4.2 Model performance

**Fig. 3:**
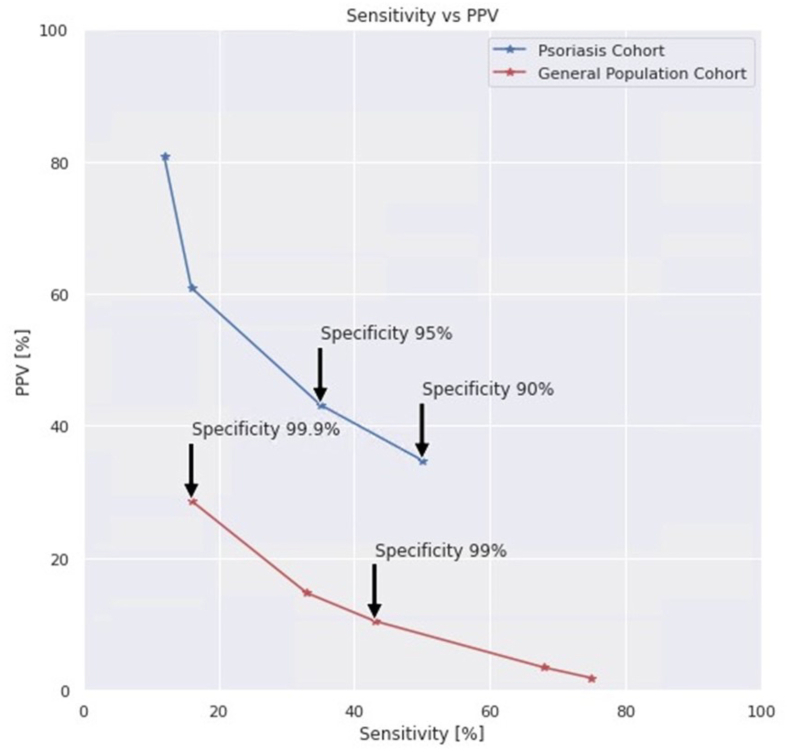
PPV vs Sensitivity, specificity cutoff selection 1 year prior to the reference event.

Four different specificity cutoffs were selected to evaluate the model. The GP cohort was evaluated at 99% and 99.9% specificities and the PsO cohort at 90% and 95% specificities. The above-mentioned cutoffs were selected for displaying practical sensitivity and PPV one year prior to the reference event as shown in the cohort PPV plot (Fig. 3).

**Fig. 4:**
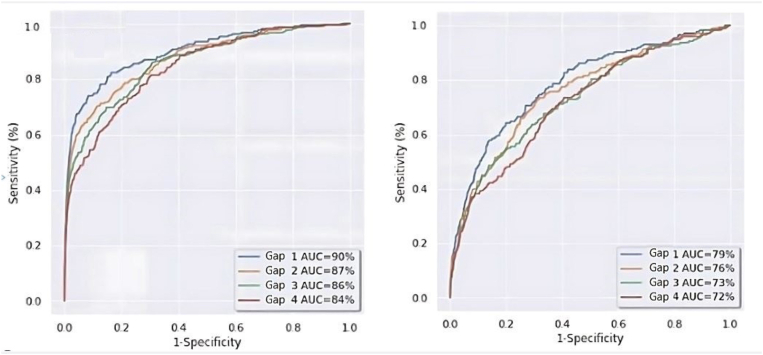
A (Left)- ROC Plot for identification of undiagnosed PsA patients in the test GP cohort, 1–4 years prior the reference event. B (Right) ROC Plot for identification of undiagnosed PsA patients in the Test PsO cohort 1–4 years prior the reference event.

The model's AUC curve for time gaps 1–4 prior to the reference event of the test GP cohort, (n = 216 PsA patients) and within the test PsO cohort (n = 174 PsA patients) are presented in Fig. 4a and b, respectively*.* The sensitivity and PPV values for identifying undiagnosed PsA patients in the GP cohort at the cut-off points of specificity 99% and 99.9% and for the PsO cohort at the cut-off points of specificity 90% and 95%, for these time gaps, are presented in Table 1, Table 2, respectively. For example, a specificity of 95% for the PsO cohort, would mean scanning an average of 2.3 PsO patients in order to identify one undiagnosed PsA patient.

**Fig. 5:**
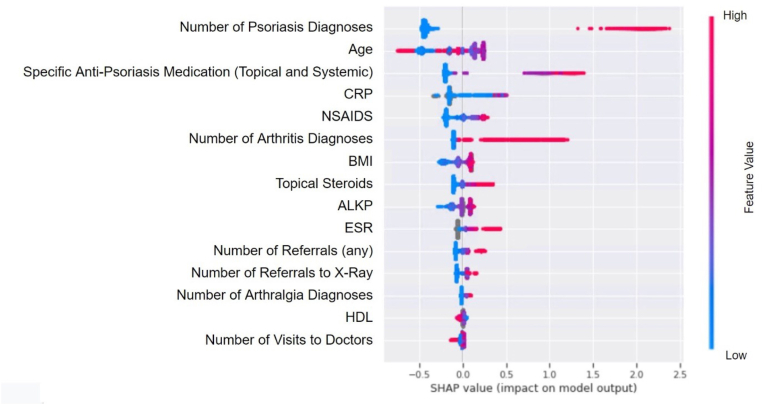
Prominent features contributing to identification of undiagnosed PsA patients within the GP cohort. The left side of the SHAP figure represents the control population and how each feature contributes to the model classifying a patient without PsA. The right side of the figure represents the case population and how each feature contributes to the model classifying a patient with PsA.

The 15 most prominent features contributing to the identification of PsA and the relationship between the value of a feature and its impact on the prediction are shown in the SHAP library in Fig. 5. The diagnosis of PsO had the highest predictive impact. Arthralgia and arthritis diagnoses also significantly contributed to the identification of undiagnosed PsA in the general population. Other high-impact factors included referrals to medical experts, hospitals and radiographic imaging, use of anti-psoriatic medications and non-steroidal anti-inflammatory drugs (NSAIDs), elevated C-reactive protein (CRP) and alkaline phosphatase (ALKP) levels.

## 5 Discussion

Early diagnosis of PsA is important since early treatment may prevent irreversible joint damage. Patient questionnaires are a useful screening and diagnostic tool. However, on-line screening tools require patient and physician involvement and are time-consuming. Third-party off-line screening, empowered by machine learning computerized algorithms, can analyze patient medical records, and save time as they do not require patient or physician involvement. Such a tool is especially important given the relatively high prevalence of undiagnosed PsA in patients with PsO [10].

In Table 1, Table 2, we present the results for higher specificity cut-off points for the GP cohort compared to the smaller PsO cohort, in order to reduce the number of false positive patients. The presented algorithm differs from other computerized algorithms designed to identify PsA patients (in contrast to undiagnosed PsA patients) [[33], [34], [35]] and which are associated with much higher PPV values. The PPV is crucial for selecting the specificity and sensitivity to use in order to optimize the costs of the diagnostic workup and screening procedures. Determining the optimal PPV cutoff is challenging, as it should be based on the prevalence of undiagnosed and not of known PsA patients. To date, only a few studies have addressed this issue. We decided to design the PsO cohort to match the 10.1% prevalence estimated by Villani et al. [10], being aware of the described limitations. Calculating the PPV when considering the prevalence of undiagnosed PsA in the general population is impossible, since no estimated undiagnosed PsA prevalence data is available within this population. Thus, before implementing the algorithm it remains to be determined whether the algorithm should be implemented in a PsO population only, or on the general population as well. In addition, the false positive rate to be used must be determined. The results in Table 1, Table 2 may support this decision-making process. The PPV values estimate how many patients were correctly identified as undiagnosed PsA patients out of those selected (flagged) by the model. By increasing specificity to 95%, sensitivity was reduced, resulting in identification of 35% of undiagnosed PsA patients. However, under these conditions, PPV increased to 43.7%, which may be more practical when implementing the algorithm on larger cohorts of patients.

Regarding the 15 parameters with the highest probability of predicting whether a patient has undiagnosed PsA (Fig. 5), the diagnosis of PsO had the highest impact. Arthralgia and arthritis diagnoses also significantly contributed to the identification of undiagnosed PsA in the general population. Other high-impact factors included referrals to medical experts, hospitals and radiographic imaging, use of anti-psoriatic medications and non-steroidal anti-inflammatory drugs (NSAIDs), and elevated C-reactive protein (CRP) and alkaline phosphatase (ALKP) levels (no available data regarding the differentiation between liver and bone ALKP). Interestingly, both CRP and ALKP [36] were recently identified as candidate biomarkers of PsA. Bone ALKP has been described as a strong potential biomarker of radiographic progression in PsA patients reflecting new bone formation [37]. In addition, an association between high liver ALKP and PsA has been recently described, especially in patients with a high body mass index (BMI) [38]. Notably, high BMI itself was identified as a high-probability impact factor by our algorithm.

We recommend continuing using questionnaires^41^ for PsA screening whenever possible. Our algorithm bears potential as a point-of-care diagnostic support tool to assist primary care providers in determining whether further workup and/or rheumatologist referral is warranted. The main limitation of the present study was the use of de-identified data which did not enable validation of PsA and PsO diagnosis via chart review. Furthermore, validating the algorithm by reviewing the EMRs to differentiate between patients who at the time gap had PsA but were not diagnosed yet and patients who did not have PsA at the time but developed PsA later on, was not possible due to restrictions of the Maccabi Institutional Review Board (IRB). Further studies will be necessary to validate this retrospectively implied algorithm for classification of true positive and false positive patients. These include similar retrospective studies on other medical databases and prospective studies.

## 6 Conclusion

PredictAI™, trained on a large community-based population dataset, may be an important point-of-care support tool to aid in early identification of undiagnosed PsA patients, in the general population, and in previously diagnosed PsO patients. Importantly, this CDS tool does not require physician or patient involvement.

To the best of our knowledge, this is one of the first reports demonstrating the use of a combined approach of gradient boosting and feature evaluation with Shapley values for the early identification of undiagnosed PsA patients. Further validation and prospective assessment of the algorithm with additional data sets is warranted.

## Authorship contributions

Jonathan Shapiro: Conceptualization, Study design, Supervision, Validation, Writing – original draft, Writing – review & editing. Benjamin Getz: Conceptualization, Data curation, Formal analysis, Methodology, Project administration, Software, Supervision, Validation, Visualization, Writing – original draft. Yonatan Jenudi: Conceptualization, Formal analysis, Software, Validation, Writing – review & editing. Michael Dreyfuss: Conceptualization, Supervision, Validation, Writing – original draft. Dan Underberger: Conceptualization, Supervision, Validation, Writing – original draft. Tahel Ilan Ber: Conceptualization, Validation, Visualization. Shlomit Steinberg-Koch: Conceptualization, Funding acquisition, Project administration, Visualization, Writing – review & editing. Amir Ben-Tov: Conceptualization, Methodology, Project administration, Supervision, Validation, Writing – original draft. Prof. Yehuda Shoenfeld: Conceptualization, Supervision, Validation, Writing – review & editing. Prof. Ora Shovman: Conceptualization, Supervision, Validation, Writing – review & editing

ALL authors approved the final version of the article, including the authorship list.

The authors would like to acknowledge Dr R Klingman and Dr. Yehudit Posen for their assistance with the manuscript editing.

## Declaration of competing interest

Shapiro J, Cohen SB, Ben-Tov A, Shoenfeld Y and Shovman O are consultants for Predicta Med Analytics Ltd. All authors declare that they have no known competing financial interests or personal relationships that could have appeared to influence the work reported in this paper.Getz B and Steinberg-Koch S have a published patent on A METHOD OF EVALUATING AUTOIMMUNE DISEASE RISK AND TREATMENT SELECTION, application number 17/596,015, however, the paper is referring to a specific use case of PsA disease.

## Data Availability

The authors do not have permission to share data.
